# 

**Published:** 1887-12-10

**Authors:** 


No. 63, Vol. III—December 10th, 1887.

[Registered at the General Post Office as a Newspaper.]

Per Annum, 6s. 6d., post free.

Monthly Parts, 6d.; post free, 7½d.

Ditto per Ann., 7s. 6d. post free.

